# Antagonistic interactions are sufficient to explain self-assemblage of bacterial communities in a homogeneous environment: a computational modeling approach

**DOI:** 10.3389/fmicb.2015.00489

**Published:** 2015-05-21

**Authors:** Román Zapién-Campos, Gabriela Olmedo-Álvarez, Moisés Santillán

**Affiliations:** ^1^Unidad Profesional Interdisciplinaria de Ingenierías Guanajuato, Instituto Politécnico NacionalSilao, Mexico; ^2^Departamento de Ingeniería Genética, Unidad Irapuato, Centro de Investigación y Estudios Avanzados del IPNIrapuato, Mexico; ^3^Unidad Monterrey, Centro de Investigación y Estudios Avanzados del IPNApodaca, Mexico

**Keywords:** bacterial antagonism, ecological modeling, community emergence, spatial patterns, bacterial biodiversity, cuatro cienegas

## Abstract

Most of the studies in Ecology have been devoted to analyzing the effects the environment has on individuals, populations, and communities, thus neglecting the effects of biotic interactions on the system dynamics. In the present work we study the structure of bacterial communities in the oligotrophic shallow water system of Churince, Cuatro Cienegas, Mexico. Since the physicochemical conditions of this water system are homogeneous and quite stable in time, it is an excellent candidate to study how biotic factors influence the structure of bacterial communities. In a previous study, the binary antagonistic interactions of 78 bacterial strains, isolated from Churince, were experimentally determined. We employ these data to develop a computer algorithm to simulate growth experiments in a cellular grid representing the pond. Remarkably, in our model, the dynamics of all the simulated bacterial populations is determined solely by antagonistic interactions. Our results indicate that all bacterial strains (even those that are antagonized by many other bacteria) survive in the long term, and that the underlying mechanism is the formation of bacterial community patches. Patches corresponding to less antagonistic and highly susceptible strains are consistently isolated from the highly-antagonistic bacterial colonies by patches of neutral strains. These results concur with the observed features of the bacterial community structure previously reported. Finally, we study how our findings depend on factors like initial population size, differential population growth rates, homogeneous population death rates, and enhanced bacterial diffusion.

## 1. Introduction

Microbial ecosystems have proved to be excellent frameworks to understanding ecological systems (Prosser et al., 2007). New ecological theories have arisen from microbial ecology due to its simplicity, controllability, replicability and experimentally-required times (Jessup et al., 2004). Remarkably, much of that progress has been achieved by means of simplified theoretical models (Momeni et al., 2011). Most of these models account for the interaction of only a few microbial populations. This is an advantage, because simple models can be more easily studied both numerically and analytically, but also a limitation, because such models oversimplify biological reality. Nowadays, thanks to the rapid increase of computer power, it is possible to investigate the dynamics of larger sets of interacting populations (Costello et al., 2012).

Organisms in an ecosystem are affected by abiotic and biotic factors. Abiotic elements refer to non-living chemical and physical factors in the environment, whereas biotic factors are living or once-living organisms in the ecosystem. The influence of abiotic factors on a community spatial-temporal dynamics has been thoroughly studied (Dunson and Travis, 1991). Conversely, biotic factors have been mostly disregarded. Nonetheless, recent studies have evidenced that biotic interaction networks play very important roles (Hooper et al., 2005), specially in terms of biodiversity maintenance and energy flow (Raes and Bork, 2008; Eisenhauer et al., 2012).

Diverse biotic interactions have been experimentally described in nature, and some others have been hypothesized from mathematical modeling results (Evans et al., 2013). At microbial scales, an outstanding panoply of interaction mechanisms has been observed (Prasad et al., 2011), favoring competition over cooperation (Foster and Bell, 2012). Antagonistic interactions (also known as interference competition) have been studied by Czárán et al. (2002), using the simplified model: Killer (K), Resistant (R) and Sensitive (S). This interaction loop implies an associated metabolic cost (and a concomitant growth-rate reduction) for being either an antibiotic producer (K) or a resistant strain (R). Thus, in terms of proliferation, S outcompetes R and R outcompetes K. This model, which is able to sustain bacterial populations, gave rise to new studies aimed at figuring out how microorganisms use and evolve bacteriocin and antagonistic molecules to sustain biodiversity in structured ecosystems (Kerr, 2007).

The shallow water system of Churince, located in Cuatro Cienegas, Mexico, sustains an impressive microorganism biodiversity, mainly due to its geological history (Souza et al., 2006). Even with scarce nutriments in the water, microorganisms have proliferated and evolved to use any available component around them, as has been seen in other oligotrophic environments (Kuznetsov et al., 1979). It is well known that some bacteria, such as *Bacillus spp*., are able to synthesize antagonistic molecules (Abriouel et al., 2011). Cuatro Cienegas' bacteria are not an exception because, as previous studies show, these organisms use a variety of bacteriocin molecules to annihilate competing neighbors (Pérez-Gutiérrez et al., 2013; Aguirre-von Wobeser et al., 2014). All of this, together with the fact that the sediment of this natural setting is static and homogeneous regarding its physicochemical conditions, make it an excellent candidate to study how biotic interactions affect the spatial-temporal arrangement of microbial populations.

In a previous study (Pérez-Gutiérrez et al., 2013), we isolated 78 bacterial strains (most of them from the genus *Bacillus*) from 5 different sampling sites across Churince pond, and tested them for one-to-one antagonistic interactions. Among others, we obtained the following results which are not self evident from the point of view of the most commonly accepted ecological theories:

- Bacterial strains are not homogeneously distributed across the pond, in spite of the pond's physical-chemical conditions being homogeneous and pretty much stationary.
- Antagonistic interactions are more frequent across sampling sites than within them.

In 2013 Pérez-Gutiérrez et al. (2013), we hypothesized that microscopic microbial antagonistic interactions may be responsible for shaping bacterial communities at the macroscopic scale, and that this may suffice to explain the above-enlisted observations. The present work is aimed at proving the feasibility of such hypothesis from a mathematical modeling perspective. To that end we decided to model the pond as a square grid, each of whose cells represents a small area that can be colonized by at most one bacterial strain. The dynamics of the grid cells are then modeled as a set of rules derived from the antagonism matrix experimentally determined in Pérez-Gutiérrez et al. (2013). We decided to employ this modeling strategy because of its adequacy given the available experimental data, and because it has been employed to demonstrate how local interactions can give rise to complicated global patterns, like those observed in natural systems (Gardner, 1970; Hogeweg, 1988; Iwasa et al., 1998; Sarkar, 2000; Wootton, 2001; Wolfram, 2002; Deutsch and Dormann, 2005). To our knowledge, this is the first study in which such a problem is tackled with an antagonistic network involving a large number of interacting strains.

## 2. Materials and methods

### 2.1. Bacterial collection

**Figure 1:**
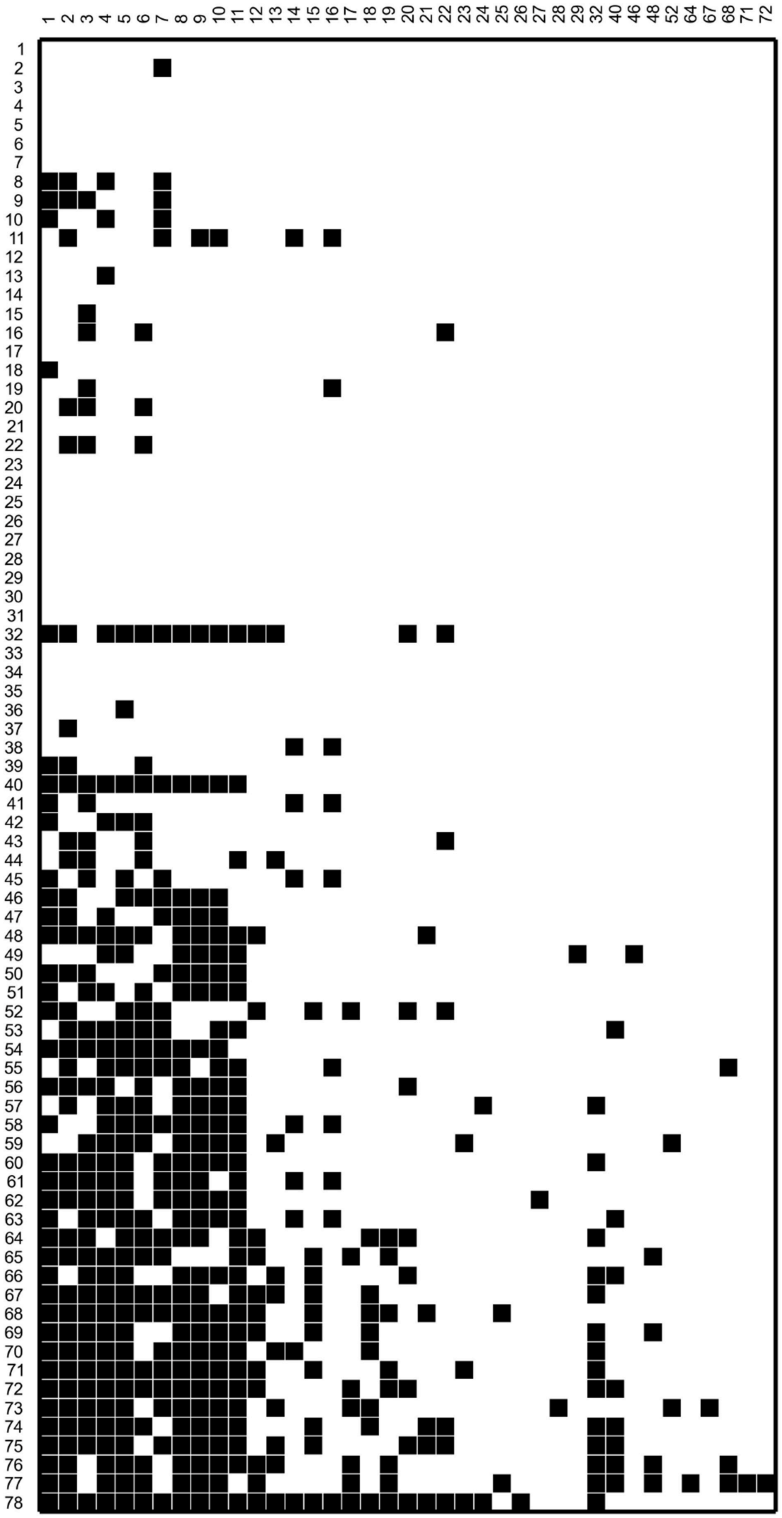
**Antagonism matrix for the bacterial strains considered in this work**. A black box indicates that the bacterial strain in the corresponding column antagonizes that in the box row. Some strains do not appear in the matrix columns because they antagonize no other bacterial strain. The bacterial strains were labeled in decreasing order according to their aggressiveness index AI (numbers of other strains antagonized by it, minus number of strains antagonizing it). In Table 1, we give the original label and the AI number of each strain.

In this work we make use of the interaction network of a set of 78 bacterial strains isolated and studied by Pérez-Gutiérrez et al. (2013). The strains in this set were isolated from 5 different samples taken from the superficial sediment of Churince pond in Cuatro Cienegas, Mexico. Since the isolating methodology involved subjecting the samples to thermal shock, all of the isolated strains came out to be thermo-resistant, and most of them belong to the genus *Bacillus*. All 78 × 78 pairs of bacterial strains were cultured in Petri dishes to test for antagonistic interactions. The resulting antagonism matrix is reported in Pérez-Gutiérrez et al. (2013) and reproduced in Figure 1. In this figure, bacterial strains are organized in decreasing order according to their *Aggressiveness Index* (number of other strains antagonized by a given strain, minus number of other strains antagonizing it). The ID numbers given in this work to all strains, the labels employed by Pérez-Gutiérrez et al. (2013), and the corresponding aggressiveness indexes are tabulated in Table 1.

**Table 1:** **ID numbers, labels, and aggressiveness indexes of the investigated bacterial strains**.

**ID**	**Label**	**AI**	**ID**	**Label**	**AI**	**ID**	**Label**	**AI**
1	CH95a	38	27	CH38c	1	53	CH44	−9
2	CH21	37	28	CH452b	1	54	CH26a	−10
3	CH90	35	29	CH159b	1	55	CH19a	−10
4	CH150a	35	30	CH39a	0	56	CH149a	−10
5	CH156	34	31	CH448a	0	57	CH155a	−10
6	CH43	31	32	CH452a	0	58	CH28	−11
7	CH22	29	33	CH99B	0	59	CH135a	−11
8	CH144a	28	34	CH88	0	60	CH98b	−11
9	CH144b	28	35	CH112a	0	61	CH84	−11
10	CH145	28	36	CH29	−1	62	CH33	−11
11	CH109a	25	37	CH93	−1	63	CH447	−12
12	CH154a	13	38	CH26b	−2	64	CH161d	−13
13	CH37	9	39	CH160c	−3	65	CH138	−13
14	CH113a	9	40	CH87b	−3	66	CH158b	−13
15	CH148	9	41	CH25	−4	67	CH145b	−14
16	CH112b	7	42	CH140a	−4	68	CH159a	−14
17	CH448b	7	43	CH111	−4	69	CH449a2	−14
18	CH23	7	44	CH157b	−5	70	CH20a	−14
19	CH20b	6	45	CH45	−6	71	CH162	−15
20	CH450	5	46	CH36	−7	72	CH446	−16
21	CH30	5	47	CH449a1	−7	73	CH40	−16
22	CH41b	4	48	CH160a	−8	74	CH445	−16
23	CH449b	3	49	CH451b	−8	75	CH451a	−17
24	CH24	2	50	CH91b	−8	76	CH163b	−17
25	CH19b	2	51	CH81a	−8	77	CH153a	−19
26	CH164b	1	52	CH142	−8	78	CH34	−26

*In the present work, bacterial strains were ordered (assigning each of them an ID number) in decreasing order according to their aggressiveness index AI (numbers of other strains antagonized by it, minus number of strains antagonizing it). We also include the label given to each strain in Pérez-Gutiérrez et al. (2013)*.

### 2.2. Computational algorithm

In order to simulate the evolution of a bacterial community interacting according to the antagonism matrix reported in Pérez-Gutiérrez et al. (2013), we developed a computational algorithm as follows:

1. A square grid of 200 × 200 cell represents the pond soil. Each cell in the grid corresponds to a small surface that can be either empty or colonized by at most one bacterial strain.
2. The grid is initialized by randomly distributing a fixed number of individual colonies of each bacterial strain among the grid cells.
3. After initializing the grid, the state of all the grid cells is updated according to the following rules:
  - Let *i* denote the current grid cell. Randomly chose one of its 8 neighboring cells and denote it by *j*.
  - If both the *i*- and *j*-th grid cells are empty at the *n*-th time step, the *i*-th cell remains empty at time *n*+1.
  - If the *i*-th grid cell is empty and the *j*-th grid cell is occupied by a given strain at time *n*, the *i*-th cell is colonized at time *n*+1 with probability *P_g_*(μ_*j*_) by the strain in the *j*-th cell, μ_*j*_. Function *P_g_*(μ*_j_*) is specified in the Results section for different simulations.
  - If the *i*-th grid cell is occupied at time *n* by strain μ*_i_*, it becomes empty at time *n*+1 with probability *P_d_*(μ*_i_*). The corresponding probability distribution is specified in the Results section for different simulations. If this does not happen, consider the following two possibilities:
    - If both grid cells (*i* and *j*) are occupied at time *n* and the strain in the *j*-th grid cell antagonizes the strain in the *i*-th grid cell, then the *i*-th grid cell gets empty at time *n* + 1.
    - If both grid cells (*i* and *j*) are occupied and no antagonism exists, or if the *i*-th grid cell is occupied and the *j*-th grid cell is empty, the *i*-th cell remains the same.
4. Step 3 is iteratively repeated a fixed number of times.

### 2.3. Generation of random and experimental-like antagonism matrices

In order to test how the system dynamics depends on the architecture of the antagonism interaction-network, we constructed alternative antagonism matrices (and employed them to repeat the analysis of the system dynamics) following two different strategies:

- Random antagonism matrices were constructed by randomly generating *N* unidirectional antagonistic interactions between the 78 isolated bacterial strains, with *N* the number of links in the original matrix. We took care of avoiding self-antagonism.
- To construct what we call experimental-like antagonism matrices, we first noted that, according to the original matrix, bacterial strains can be classified as high-level, medium-level and low-level antagonistic strains (a k-means algorithm, MacQueen, 1967 was employed to carry out this classification). Then, we made use of a Markov-Chain Monte Carlo algorithm (Gilks, 2005) to randomly generate *N* links in such a way that the resulting antagonism matrix has the same number of high-level, medium-level, and low-level antagonistic strains as the original matrix.

## 3. Results

### 3.1. Emergency of community patches provides a survival mechanism to low-level antagonistic bacteria

**Figure 2:**
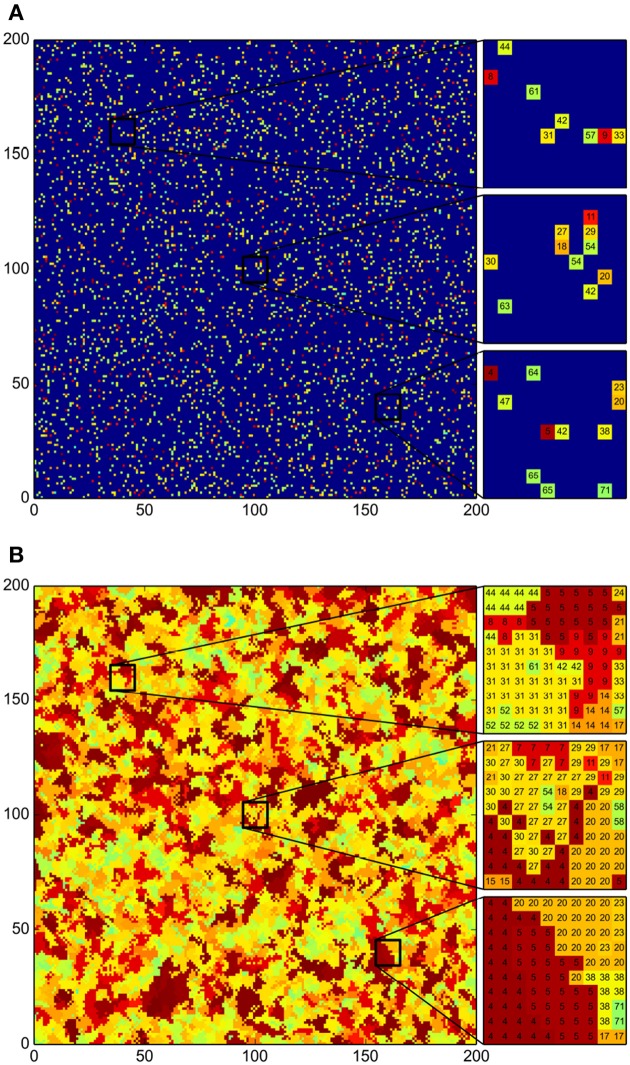
**Emergence of community patches as a result of bacterial growth**. Color indicates strain *Aggressiveness Index* (*AI*). The less aggressive bacteria are represented in light green and the most aggressive ones in maroon; deep blue denotes an empty grid cell. The *AI* of the *i*-th bacterial strain is computed as the number of other strains antagonized by it minus the strain count that antagonize it. **(A)** Initial distribution of the 78 bacterial strains. For each strain 50 initial individual colonies were distributed randomly. **(B)** Final distribution after 500 iteration steps of the simulation algorithm. Total coverage of the surface and emergence of bacterial patches isolating populations can be observed. Beside both graphs we present close-ups of selected regions across the grid, with the ID number of the strain inhabiting each grid cell indicated within (see Table 1).

To investigate whether, as claimed by Pérez-Gutiérrez et al. (2013), antagonistic interactions have a lead role in the spatial distribution of bacterial communities, as well as in the diversity differences found across sites, we took the antagonism matrix reported therein, and used it to implement the algorithm described in the Section 2. Recall that, in such algorithm, a 200 × 200 square grid represents the pond, and each cell in the grid corresponds to a small surface in the pond soil that can either be empty or colonized by at most one strain. Initially, the grid was “inoculated” with 50 individual colonies of every bacterial strain, randomly distributed across the grid (see Figure 2A). That is, the grid was seeded with 78 × 50 = 3900 cells in total. The system was then iteratively evolved in time for 500 round cycles of the algorithm. In these initial simulations, we assumed for all strains (μ) that the probability that the bacterial strain in a grid cell colonizes a neighboring empty cell in an algorithm step is 1 (*P_g_*(μ) = 1), and that the probability that an inhabited grid cell is emptied because the corresponding colony dies is zero (*P_d_*(μ) = 0). To clarify the role of antagonism in the evolution of the system, an aggressiveness index was defined as follows: the aggressiveness index of a given bacterial strain, *AI*(μ), equals the number of other strains antagonized by it, minus the number of other strains that antagonize it.

In Figure 2 we present the results corresponding to a single simulation. We carried out several simulations and obtained equivalent results in all cases. Namely, an emergent spatial arrangement of bacterial communities in patches spans the grid after a few iteration steps, and eventually a stationary state in which the patches no longer change is reached (see Figure 2B). We note that the formation of patches is consistent with the fact that different strains were isolated from different samples (Pérez-Gutiérrez et al., 2013), despite the habitat homogeneity. Moreover, we can see in this particular simulation, but this is a consistent observation, that patches of vulnerable strains are always surrounded, and shielded from the most aggressive strains, by patches of other strains that are both resistant to the aggressive ones and non-antagonistic toward vulnerable bacteria. This result suggests a mechanism for the survival of low-antagonistic and vulnerable bacteria, but also offers a plausible explanation for the observation that antagonistic interactions within a sampling site are on average less frequent than interactions across sites (Pérez-Gutiérrez et al., 2013).

**Figure 3:**
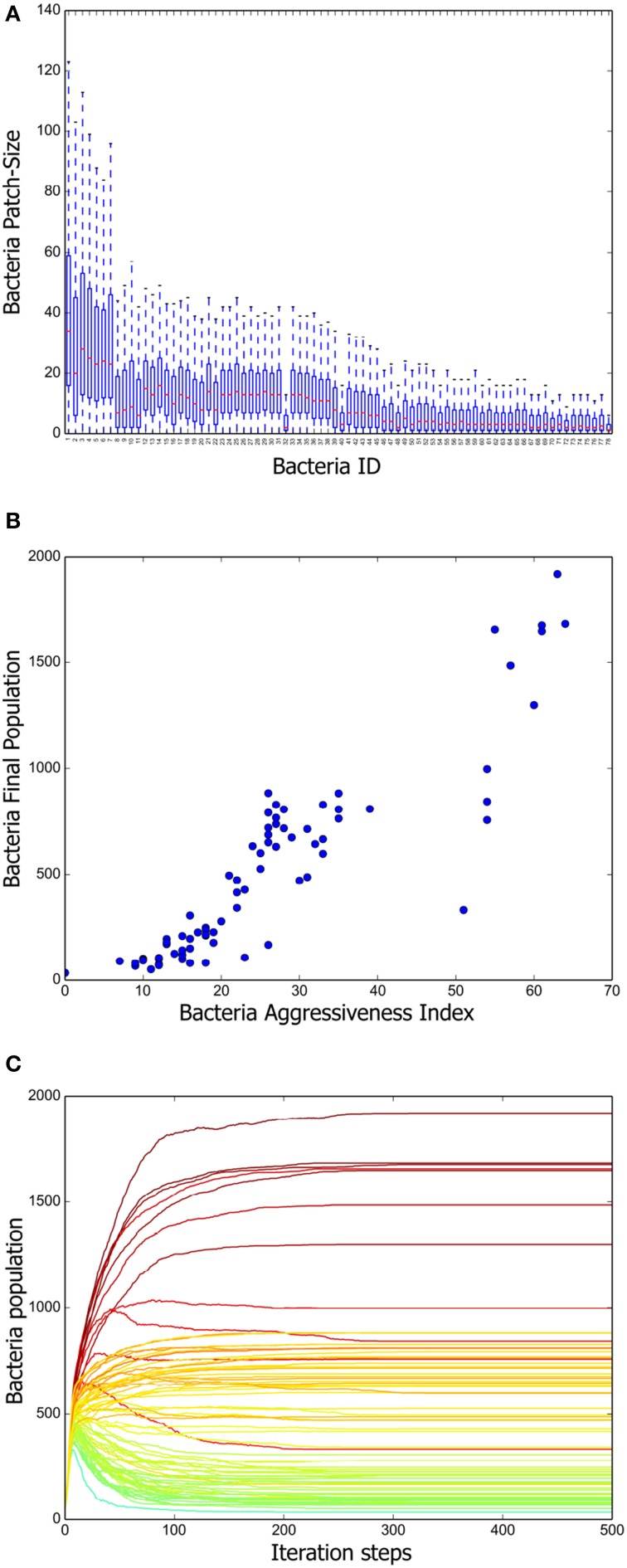
**Positive long-term correlation between *AI* and bacterium population. (A)** Box plot summarizing the patch-size statistics of 100 simulations. Bacterial strains are shown in decreasing order according to their AI value. Note that a positive correlation exists between aggressiveness index and average patch size. **(B)** Correlation between stationary bacterial populations, averaged over 100 simulations, and *AI* values. A constant was added to the previously defined *AI* so that it attains positive values for all strains. **(C)** Growth curves for all the bacterial strains tabulated in Table 1. The AI value corresponding to each strain is indicated by means of the same color code as in Figure 2. Notice that, initially, all strains populations increase monotonically. However, once the grid becomes saturated, antagonistic interactions make the population of low *AI* strains decrease.

To have a deeper understanding of the system dynamics we measured patch sizes in the stationary state of 100 different simulations. The results are summarized in Figure 3A. We can appreciate there that a positive correlation exists between the mean patch size and the aggressiveness index of a given strain. That is, more aggressive bacteria tend to form larger patches, whereas the patches of more vulnerable bacteria are consistently smaller. We further measured the total population of every strain at the end of 100 different simulations and computed the corresponding averages. A scatter plot representing mean final population size vs. aggressiveness index is shown in Figure 3B. Observe that total final population is also positively correlated with the aggressiveness index.

Finally, we followed the evolution in time of all the strain populations and the results (averaged over 100 different simulations) are shown in Figure 3C. Observe that, initially, all the inoculated colonies grow steadily, but eventually, a stratification of them according to their *AI* value becomes apparent. As far as we understand, this happens because reduction of the distance between colony borders increases the frequency of conflicts. After about 200 simulation steps, a stationary regime, in which the population size of all bacteria strains remains constant, is reached.

### 3.2. Bacterial-community spatial structure is disrupted under constant perturbation and all vulnerable strains are driven to extinction

**Figure 4:**
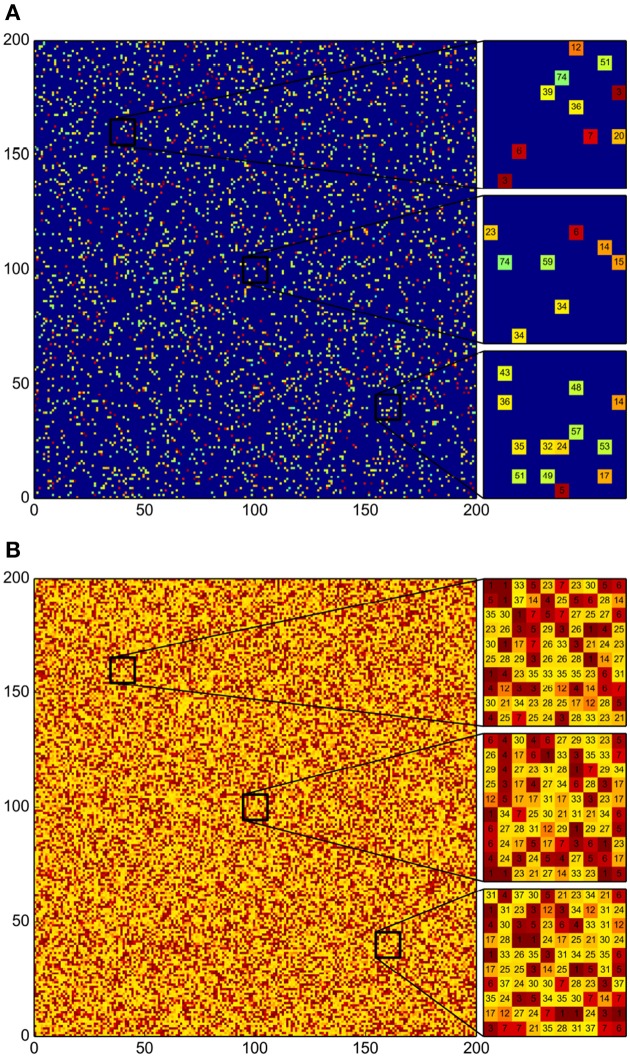
**Disruption of the emergent community patches by regularly shuffling of the grid. (A)** Initial distribution of the 78 bacterial strains. For each strain 50 initial individual colonies were distributed randomly. **(B)** Final distribution after 500 iteration steps of the simulation algorithm. Every 10 iterations the grid was randomly shuffled. As in the simulations without shuffling, the grid ends up being completely occupied. However, a structure without patches emerges, and all susceptible bacterial strains are missing.

**Figure 5:**
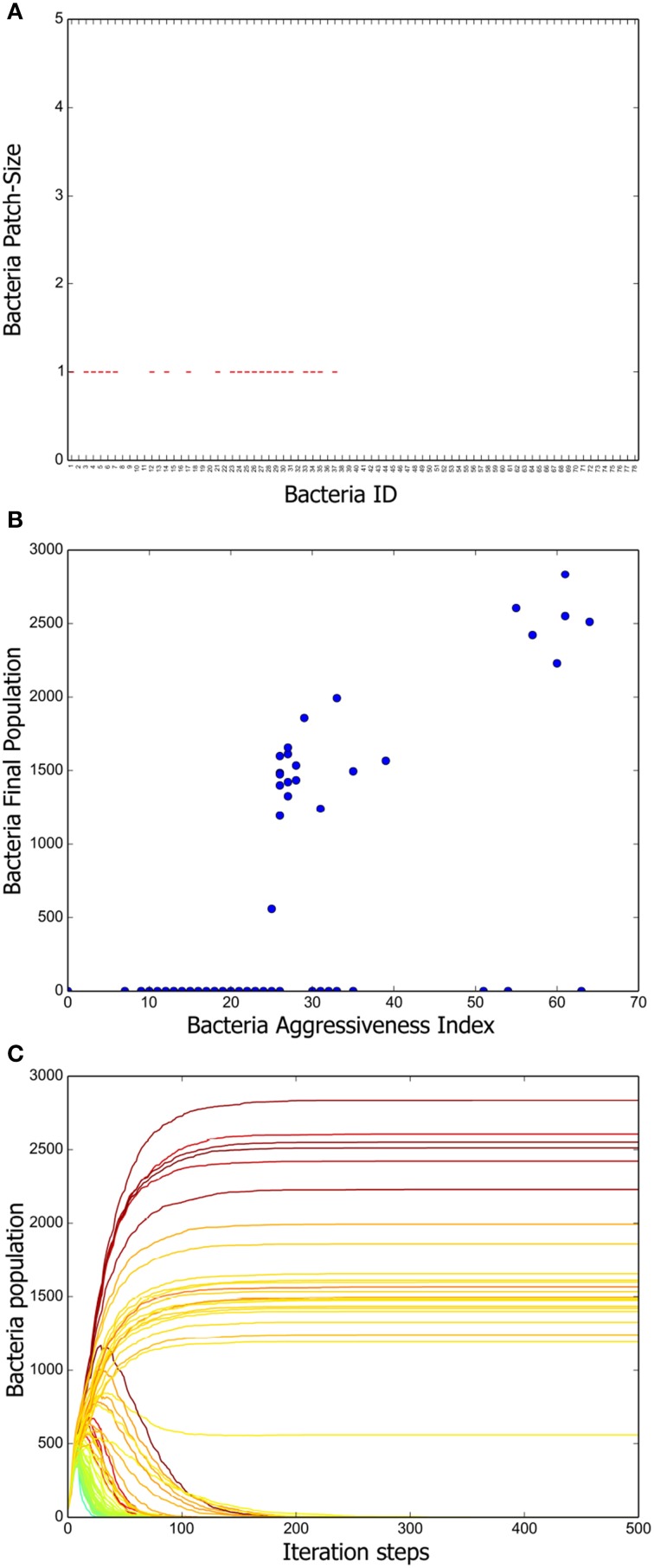
**Susceptible strains become extinct due to regular strain shuffling. (A)** Box plot summarizing the patch-size statistics of 100 simulations. Bacterial strains are shown in decreasing order according to their AI value. Many strains become extinct and the patch size for all the surviving-strain colonies is exactly 1. That is, no patches are formed whatsoever. **(B)** Correlation between *AI* and stationary population levels. Observe that all susceptible strains (most of them with low antagonism levels) become extinct and that the surviving strains occupy the space left by those that become extinct. **(C)** Growth curves for all bacterial strains in this Figure. Notice that regular reshuffling of the grid does not affect bacterial population levels once a stationary state has established.

Since the spatial arrangement of bacteria in stagnant community patches appeared to be necessary for the preservation of bacterial diversity, we carried out an alternative experiment with constant shuffling (mixing) of the grid cells. In these experiments, we carried out simulations as previously described (see Figure 4A), but every 10 simulation steps the grid cells were randomly shuffled (different inter-shuffling times were also considered, but all of them led to equivalent results). We observed that all susceptible bacterial strains (most of them with low antagonistic levels) become extinct, and they are replaced by larger populations of medium- and high-level antagonist strains (see Figures 5B,C). Furthermore, the surviving bacteria do not form patches. Instead, single-grid-cell colonies are randomly distributed (in a well mixed fashion) across the grid (see Figures 4B, 5A). Even though we shuffled the grid at regular times, the first shuffling events have the most notorious effects: they rapidly cause the extinction of the sensitive strains. After this, the populations of surviving bacteria reach a stationary value, independently of the changing spatial distribution (see Figure 5C). A correlation between stationary populations and *AI* values is still present for the surviving strains (see Figure 5B).

The above results indicate, to our consideration, that periodic shuffling of the grid cells not only precludes patch formation (by randomly relocating individual colonies of all strains) but also ensures that all susceptible strain colonies eventually get in contact with aggressive strains and are thus driven to extinction.

### 3.3. Influence of the antagonism matrix architecture on the system dynamic behavior

To answer the question of whether the results discussed in previous subsections depend on the architecture of the antagonistic-interaction network (which is determined by the antagonism matrix), we generated random and experimental-like interaction matrices (see Section 2). Experimental-like interaction matrices have the same number of high-level, medium-level, and low-level antagonistic strains, and the distribution of exerted-antagonism and received-antagonism links are very similar to those of the experimentally obtained matrix. On the other hand, random antagonism matrices were built by linking, via antagonism interactions, couples of bacterial strains chosen at random (avoiding self-antagonism). The number of antagonism links in the random and the experimentally-obtained matrices are always the same.

When we repeated the simulations using experimental-like interaction matrices, we were able to recover all the previously described results, with and without shuffling. Nonetheless, the simulations with random interaction matrices rendered quite different results. Community patches can still be observed in the non-shuffling simulations, but the final bacterial population size is less disperse than in the simulations carried out with the experimentally-obtained or the experimental-like antagonism matrices. Furthermore, in the shuffling simulations, most bacterial strains become extinct and no correlation is observed between *AI* and the stationary population size (see Supplementary Material).

In conclusion, the architecture of the interaction network plays a very important role in the emergence of the community patch structure. In particular, the characteristic that seems to be essential is that bacterial strains can be classified in three different classes: aggressive (highly antagonistic and resistant to other strains), neutral (barely antagonistic and resistant to aggressive strains), and vulnerable (non-antagonistic and sensitive to aggressive strains).

### 3.4. Influence of growth rate and initial population size on the system dynamics

So far, we have assumed in our simulations that all bacterial strains grow at the same rate, whenever they have available space. Recall that in our model the growth rate of strain μ is determined by probability *P_g_*(μ). Moreover, we considered equal initial populations in all of our simulations. In order to have a more complete picture of the studied biological phenomena, we repeated our simulations by taking into consideration distinct growth probabilities and initial populations.

Based on previously reported observations (Bohannan et al., 2002; Kneitel and Chase, 2004; Cadotte, 2007), who have shown that changes in the flow of metabolic energy and in the cellular machinery need to be done by a bacterium in order to become either highly antagonist or fast growing, we assumed that *P_g_*(μ) is inversely proportional to *AI*(μ) (the strains with the largest and the smallest *AI* values have *P_g_* = 1 and *P_g_* = 0.5, respectively), and repeated the previously described simulations. According to our results, the new simulations differ from the previous ones in that low-level and medium-level antagonistic strains have now larger stationary populations, at the expense of high-level antagonistic strains (see Supplementary Material).

To account for variable initial populations, we took the final population distributions of the simulations described in Section 3.1, and used them to compute the initial strain populations of a new grid, with the constraint that the most populous strain initially occupies 1% of the grid cells. After running the simulations, the only notorious difference we observed, with respect to those corresponding to constant initial populations, is that having larger initial population has a positive effect on the corresponding stationary population. This happens regardless of whether we consider constant or variable growth rates (see Supplementary Material).

### 3.5. Non-zero death rate may increase the chance of survival of less antagonistic bacteria

Finally, we considered in our model a death rate related to causes independent of bacterial antagonism. According to Servais et al. (1985); Pace (1988), in aquatic environments there are several events that generate the reduction of certain populations of bacteria, due primarily to: biotic factors (viral infections, starvation, senescence, and allelopathy) and abiotic factors (environmental changes such as radiation, temperature changes and toxic compounds). To take this into account we assumed that, in every step of the algorithm, a grid cell inhabited by strain μ has a probability *P_d_*(μ) of being emptied, independently of growth and antagonism events.

Initially, we considered a constant *P_d_*(μ) = 0.01 for all strains. With this assumption, we were able to recover all the results described in previous sections, with the exception that the most favored strains (those with the largest stationary populations) are those with intermediate *AI* values. I.e., those strains that neither antagonize many other bacteria nor are susceptible to be antagonized by the most aggressive ones (see Supplementary Material). When we repeated the simulations with *P_d_*(μ) = 0.1, we obtained similar results, except that the strains that had the smallest stationary populations in the previous simulations were driven to extinction (see Supplementary Material). Interestingly, when a non-zero death rate is accounted for, the achieved stationary state is not stagnant any longer. Community patches are formed, but they do not remain the same once the stationary population levels are achieved and the grid is full. On the contrary, they slowly change their size and shape, and move around the grid.

## 4. Discussion and conclusions

The computational modeling framework employed in this work has proved to be very useful to studying the spatial and temporal behavior of diverse biological phenomena (Ermentrout and Edelstein-Keshet, 1993; Alber et al., 2003; Rohde, 2005). Hence, we decided to employ it to test the hypothesis that macroscopic community patches can emerge as the result of microscopic individual interaction in a homogeneous environment. Our results not only confirm the feasibility of such hypothesis, but also show that these patches allow bacteria to minimize conflicts while preserving biodiversity.

We tested the robustness of our results by considering different initial-condition scenarios, as well as non-zero death rates and distinct growth rates for different bacterial strains. In all cases we obtained qualitatively equivalent results, thus confirming that the achieved conclusions do not depend on these factors.

On the other hand, it must be emphasized that the rules underlying the implemented computational algorithm are concomitant with low-motility bacteria growing in a homogeneous surface, interacting through pairwise-local-interactions. These restrictions are consistent with the environmental conditions observed in Cuatro Cienegas ponds (Johannesson et al., 2004), where oligotrophic constant conditions have given rise to a great diversity of bacteria who take advantage of any component in the media around them (Escalante et al., 2008; Cerritos et al., 2011) and compete with direct neighbors (Pérez-Gutiérrez et al., 2013; Aguirre-von Wobeser et al., 2014). However, we wondered to what extent having low-motility bacteria is a necessary condition for biodiversity preservation. To test this, we repeated our simulations but included periodical and random shuffling of the grid cells. Since we invariably observed that all susceptible strains become extinct, we conclude that having a slowly changing environment is mandatory for sustaining biodiversity when highly antagonistic, neutral, and highly susceptible strains share the ecosystem.

Previous studies have shown the importance of biodiversity in food-webs, being the web architecture the cause and effect of biodiversity prevalence (Sole and Montoya, 2001; Dunne et al., 2002; Ives and Carpenter, 2007; Allesina and Pascual, 2008), specially under perturbation scenarios (Girvan et al., 2005; Pascual and Guichard, 2005; Dunne and Williams, 2009; Baho et al., 2012). Counter-intuitively our results suggest that antagonism interaction networks may have a similar effect. Previous similar studies have been published (Silvertown et al., 1992; Kerr et al., 2002; Kirkup and Riley, 2004; Károlyi et al., 2005; Walshe, 2006), and some report a large repertoire of possible dynamic behaviors (Silvertown et al., 1992; Károlyi et al., 2005). However, to our knowledge, this is the first study in which a large set of experimental antagonism data is considered.

We are conscious that the model here introduced does not provide a detailed picture of the real-life system. Instead, it can best be regarded as a very simple cartoon or toy model. This is so because the model ignores dynamic aspects that play important ecological roles. For instance, positive interactions are well-documented in the case of biofilms, which tend to aggregate various types of bacteria and promote positive interactions among them (in this way, positive interactions can generate micro-habitats which introduce a level of physicochemical heterogeneity even in an otherwise rather stable and constant habitat). In this respect, we build our model following Einstein's advice that every theory (model) should be as simple as possible, but not simpler (i.e., not so simple that it does not represent reality any longer). To our consideration, given the amount of available experimental information, the present is the simplest possible model one can come out with to tackle the question of whether the antagonism matrix found by Pérez-Gutiérrez et al. (2013) can explain a heterogeneous bacterial community distribution in a homogeneous habitat.

## Author contributions

GO and MS conceived the project, designed the *in-silico* experiments, and interpreted data and results. RZ conducted the experiments and analyzed and interpreted data. All authors contributed to writing the manuscript.

### Conflict of interest statement

The authors declare that the research was conducted in the absence of any commercial or financial relationships that could be construed as a potential conflict of interest.
