# Supplementary material for: Antagonistic interactions are sufficient to explain self-assemblage of bacterial communities in a homogeneous environment: a computational modeling approach
Source: Front Microbiol. 2015 May 21;6:489. doi: 10.3389/fmicb.2015.00489 (PMC4440403; doi:10.3389/fmicb.2015.00489)
Supplement: Supplementary file 1 [file Presentation1.PDF]

Frontiers in Microbiology

Research Article
23 February 2015

Supplementary Material
Antagonistic interactions are sufficient to explain self-assemblage of bacterial communities in a homogeneous environment: a computational modeling approach

Román Zapién-Campos [1], Gabriela Olmedo-Álvarez [2] and Moisés Santillán [3,*]

[1] *UPIIG, Instituto Politécnico Nacional, Silao, México*
[2] *Departamento de Ingeniería Genética, Unidad Irapuato, CINVESTAV, Irapuato, México*
[3] *Unidad Monterrey, CINVESTAV, Apodaca, México*

Correspondence*:
Moisés Santillán
Unidad Monterrey, CINVESTAV, Vía del Conocimiento 201, Parque PIIT, Apodaca, 66600 México, msantillan@cinvestav.mx

Conflict and Cooperation in Microbial Societies

1 SUPPLEMENTARY FIGURES

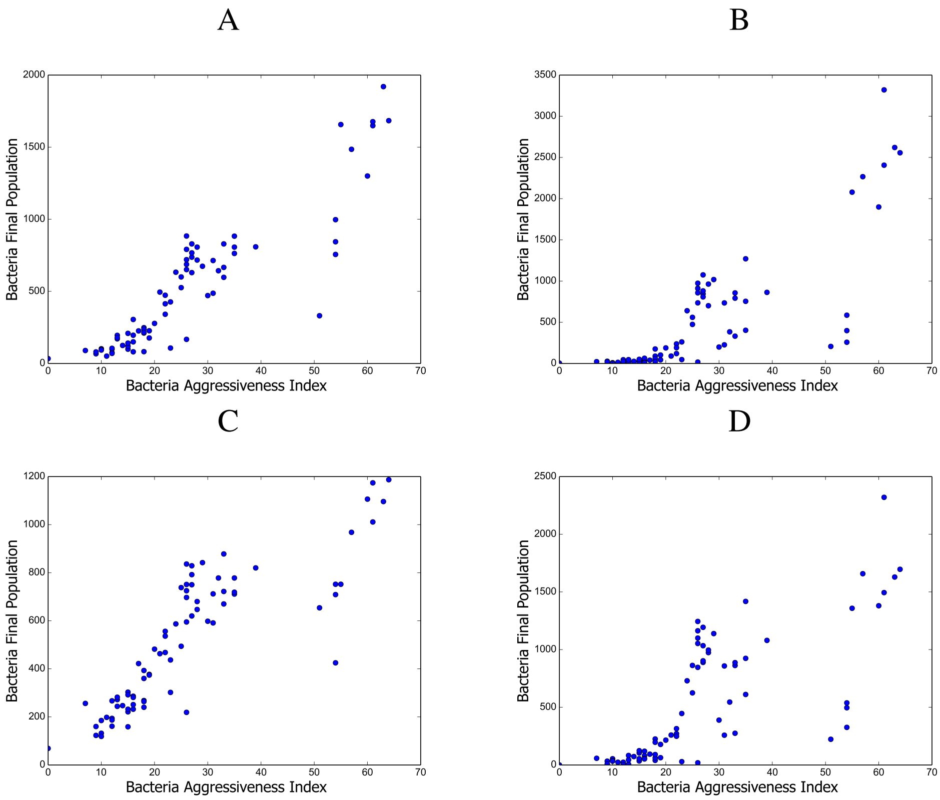

Figure 1. Plots of stationary bacterial populations vs. aggressiveness index resulting from simulations in which A) uniform initial populations and growth rates, B) varying initial populations and uniform growth rates, C) uniform initial populations and varying growth rates, and D) varying initial populations and growth rates were considered. See the main text for further details.

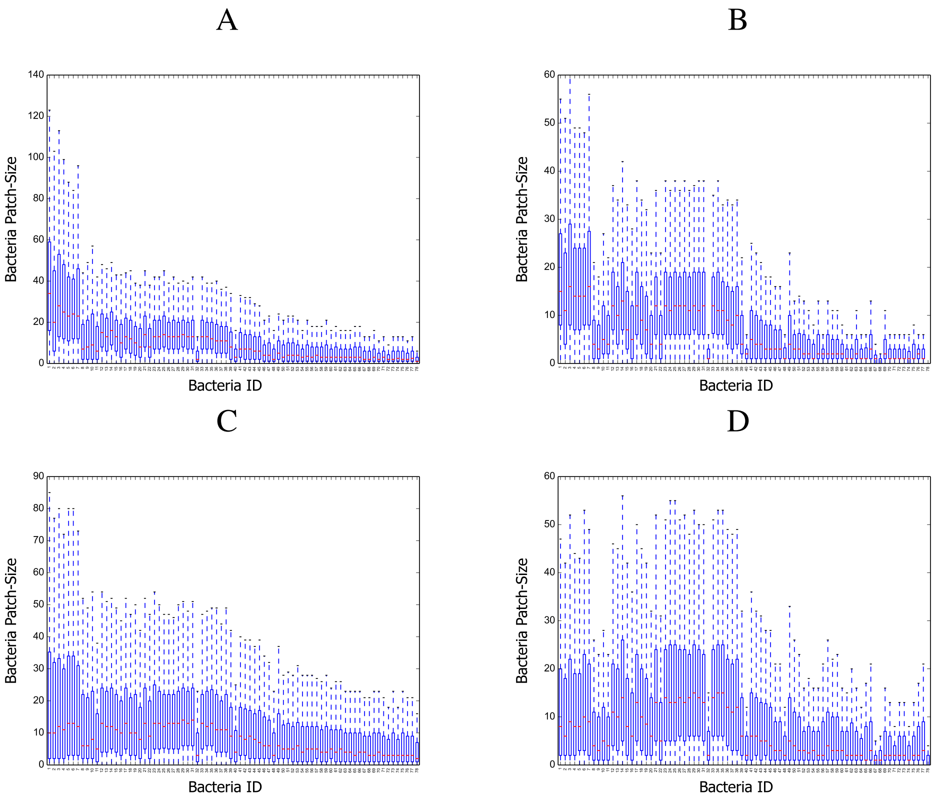

Figure 1. Box-plot graphics summarizing the patch-size statistics of 100 simulations carried out in the same conditions as the corresponding graphics in Fig. 1. Bacterial strains are shown in decreasing order according to their AI value. See the main text for further details.

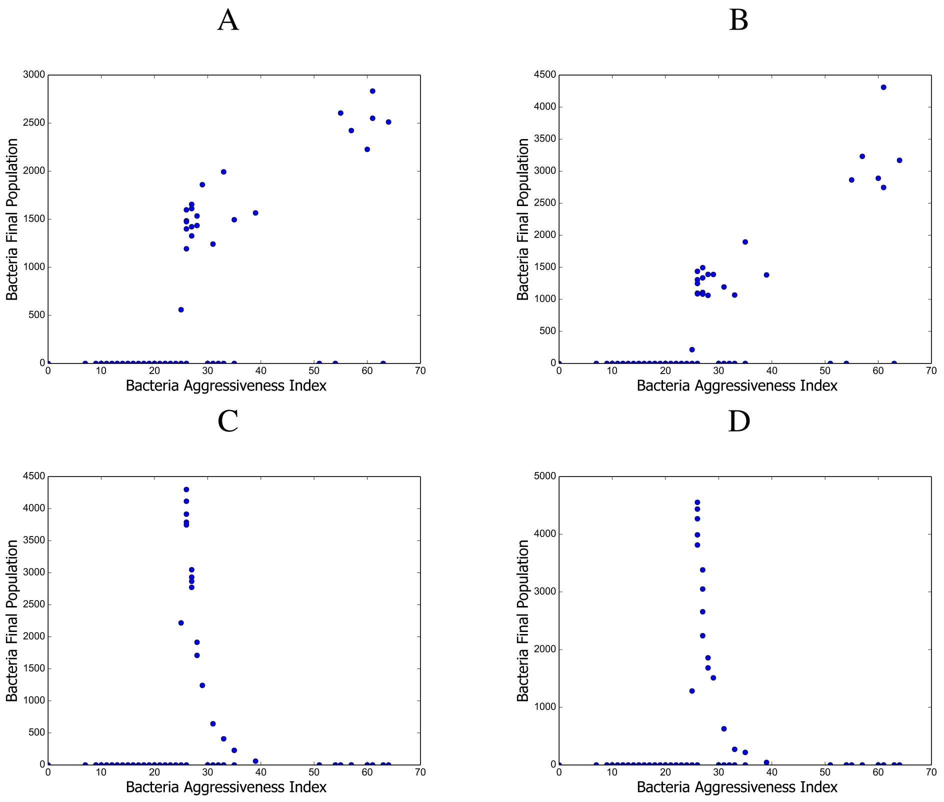

Figure 2. The same as in Fig. 1, but with the addition of random shuffling of the grid cells every 10 simulation steps.

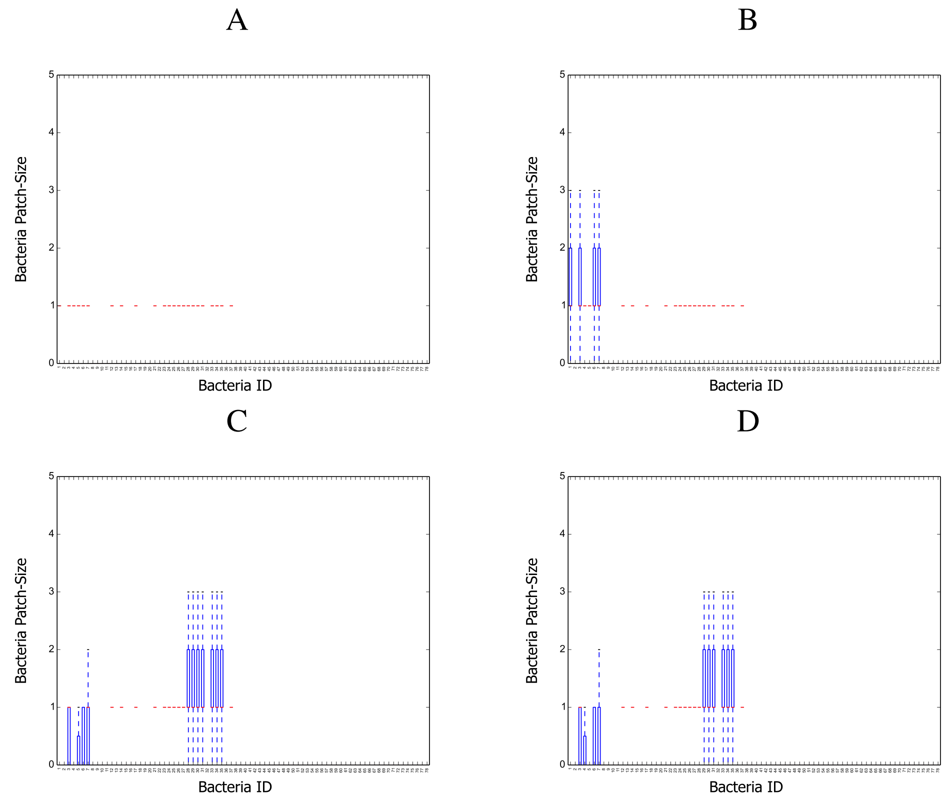

Figure 2. Box-plot graphics summarizing the patch-size statistics of 100 simulations carried out in the same conditions as the corresponding graphics in Fig. 2. Bacterial strains are shown in decreasing order according to their AI value. See the main text for further details.

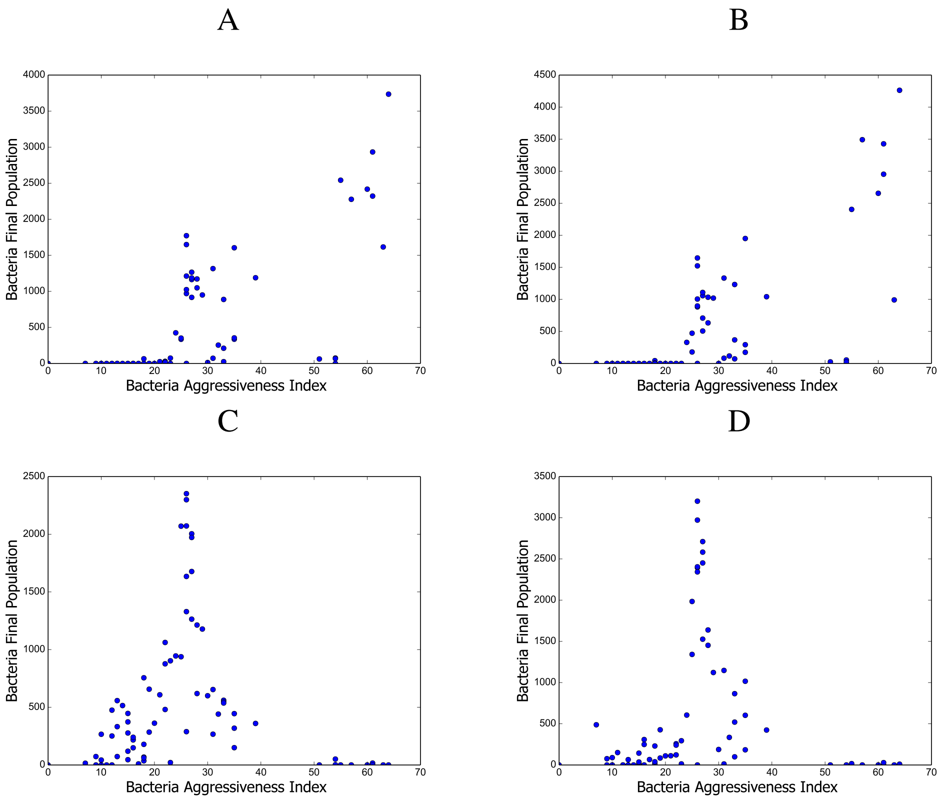

Figure 3. The same as in Fig. 1, but considering $P_d(\mu) = 0.01$ for all strains.

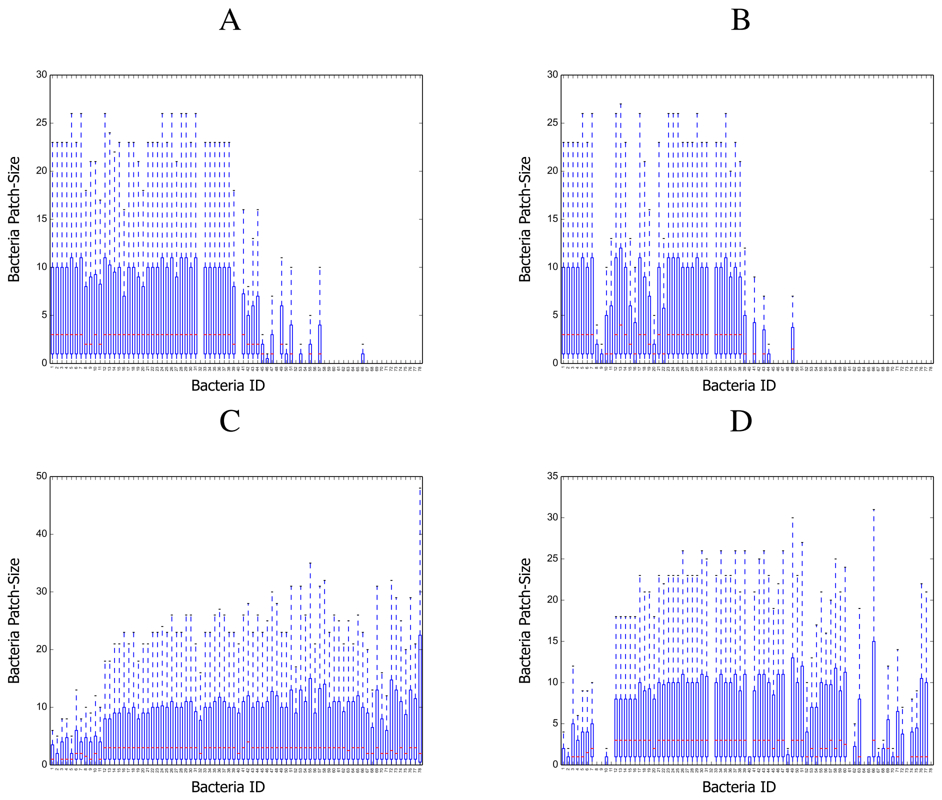

Figure 3. Box-plot graphics summarizing the patch-size statistics of 100 simulations carried out in the same conditions as the corresponding graphics in Fig. 3. Bacterial strains are shown in decreasing order according to their AI value. See the main text for further details.

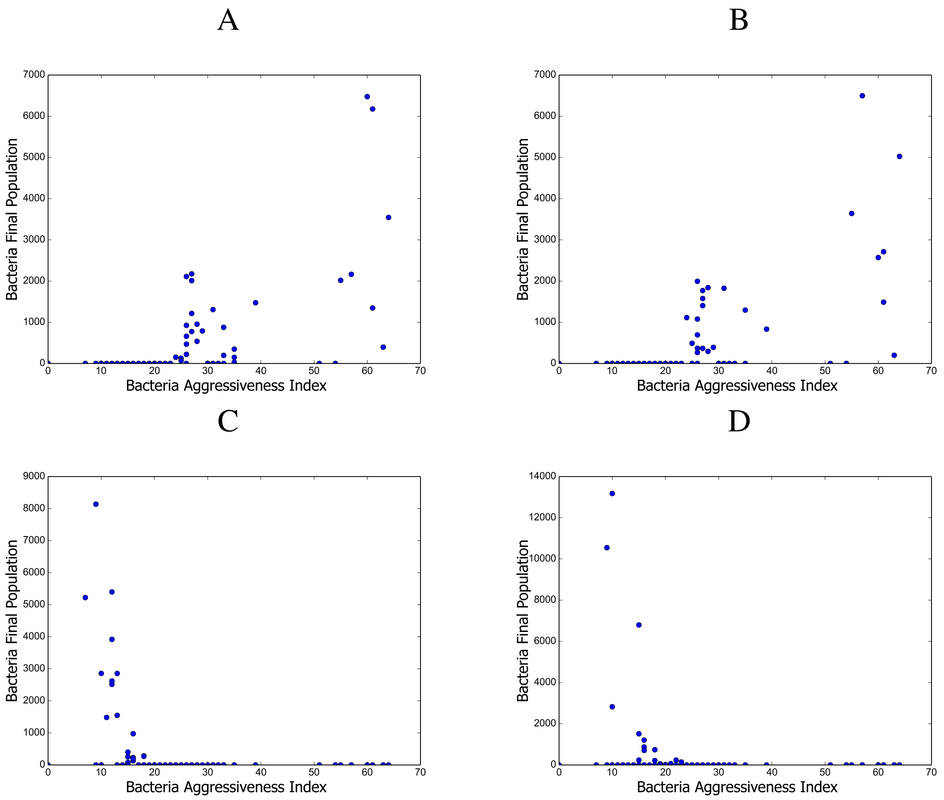

Figure 4. The same as in Fig. 1, but considering $P_d(\mu) = 0.1$ for all strains.

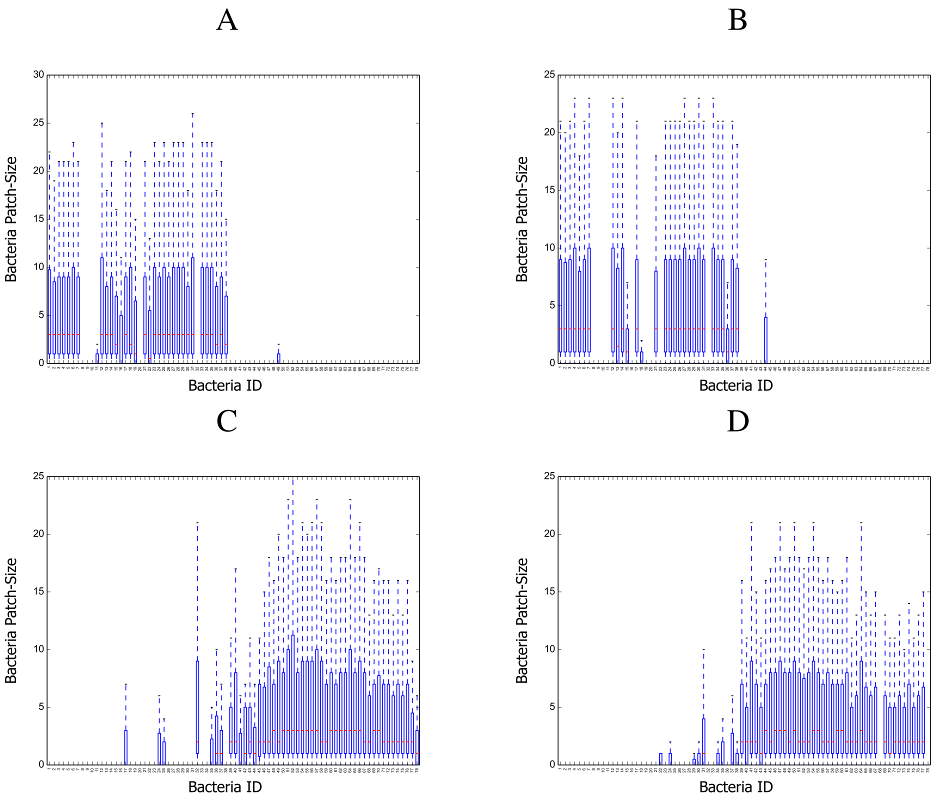

Figure 4. Box-plot graphics summarizing the patch-size statistics of 100 simulations carried out in the same conditions as the corresponding graphics in Fig. 4. Bacterial strains are shown in decreasing order according to their AI value. See the main text for further details.

A

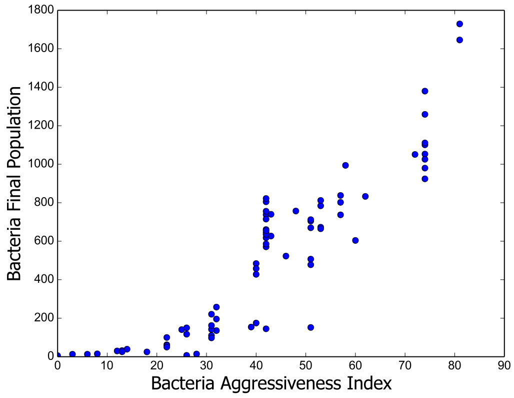

B

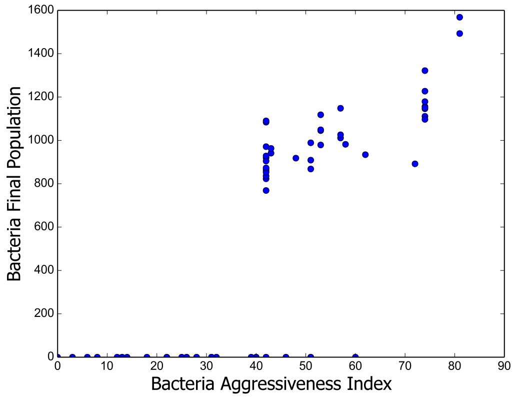

Figure 5. Plots of stationary bacterial populations vs. aggressiveness index using an experimental-like antagonism matrix when A) the grid cells are static, B) the grid cells are shuffled every 10 simulation steps randomly. See the main text for further details.

A

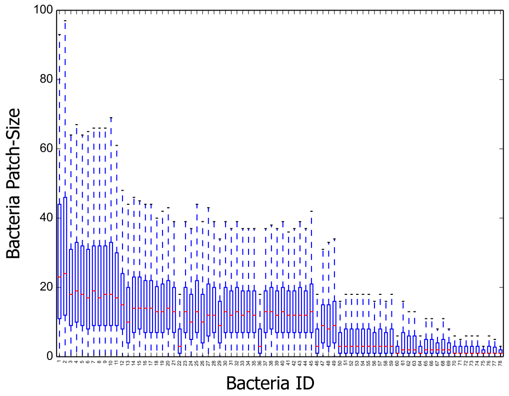

B

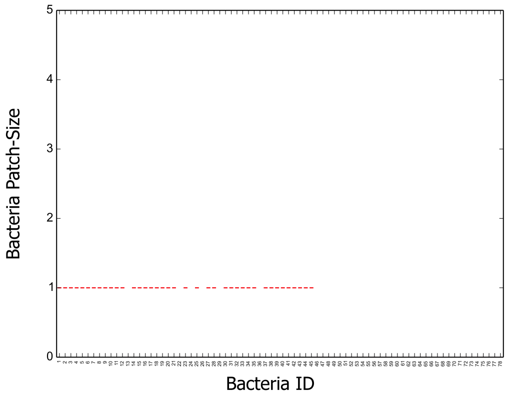

Figure 5. Box-plot graphics summarizing the patch-size statistics of 100 simulations carried out in the same conditions as the corresponding graphics in Fig. 5. Bacterial strains are shown in decreasing order according to their AI value. See the main text for further details.

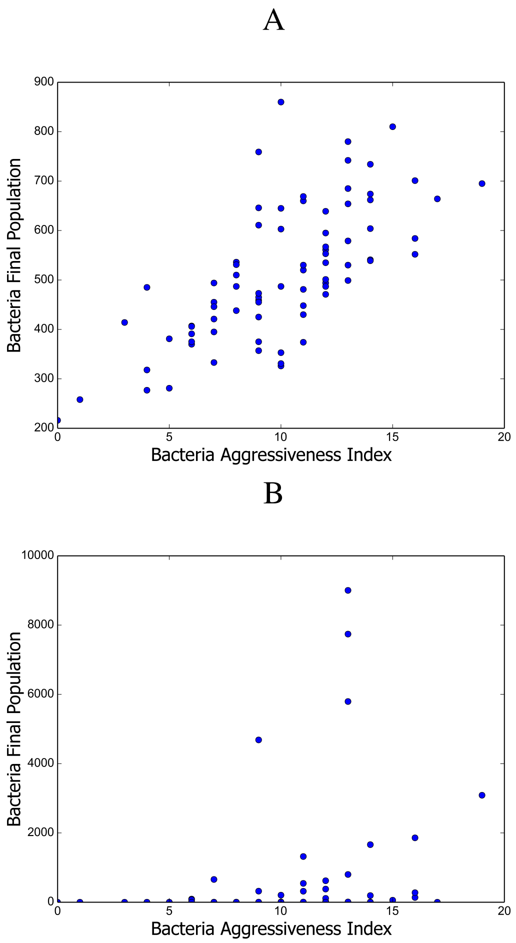

Figure 6. Plots of stationary bacterial populations vs. aggressiveness index using a random antagonism matrix when A) the grid cells are static, B) the grid cells are shuffled every 10 simulation steps randomly. See the main text for further details.

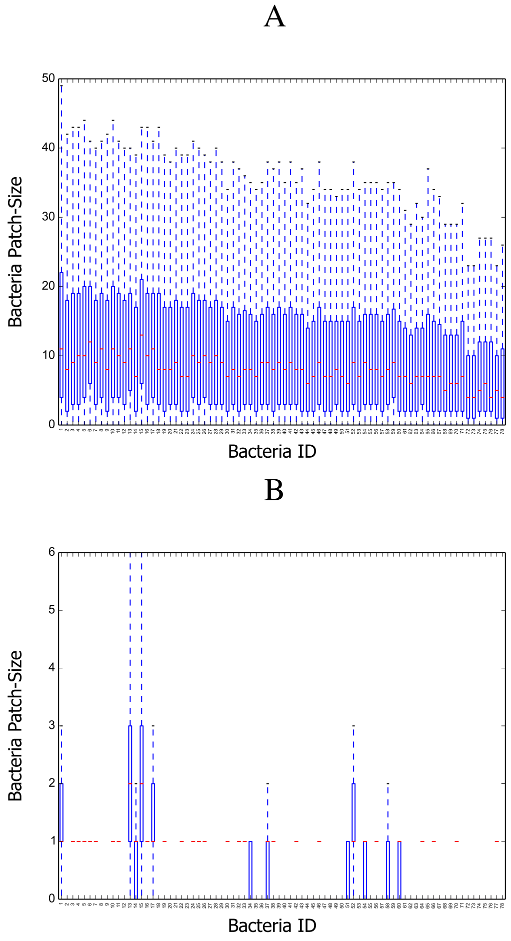

Figure 6. Box-plot graphics summarizing the patch-size statistics of 100 simulations carried out in the same conditions as the corresponding graphics in Fig. 6. Bacterial strains are shown in decreasing order according to their AI value. See the main text for further details.
